# Machine learning survival prediction using tumor lipid metabolism genes for osteosarcoma

**DOI:** 10.1038/s41598-024-63736-y

**Published:** 2024-06-05

**Authors:** Shuai Li, Zhenzhong Zheng, Bing Wang

**Affiliations:** Department of Spine Surgery, The Second Xiangya Hospital, Central South University, Renmin Middle Road 139, Changsha, 410011 Hunan China

**Keywords:** Osteosarcoma, Lipid metabolism, Molecular subtypes, Survival prediction, Signature, Cancer genetics, Cancer metabolism, Cancer microenvironment, Gene ontology, Machine learning

## Abstract

Osteosarcoma is a primary malignant tumor that commonly affects children and adolescents, with a poor prognosis. The existence of tumor heterogeneity leads to different molecular subtypes and survival outcomes. Recently, lipid metabolism has been identified as a critical characteristic of cancer. Therefore, our study aims to identify osteosarcoma's lipid metabolism molecular subtype and develop a signature for survival outcome prediction. Four multicenter cohorts—TARGET-OS, GSE21257, GSE39058, and GSE16091—were amalgamated into a unified Meta-Cohort. Through consensus clustering, novel molecular subtypes within Meta-Cohort patients were delineated. Subsequent feature selection processes, encompassing analyses of differentially expressed genes between subtypes, univariate Cox analysis, and StepAIC, were employed to pinpoint biomarkers related to lipid metabolism in TARGET-OS. We selected the most effective algorithm for constructing a Lipid Metabolism-Related Signature (LMRS) by utilizing four machine-learning algorithms reconfigured into ten unique combinations. This selection was based on achieving the highest concordance index (C-index) in the test cohort of GSE21257, GSE39058, and GSE16091. We identified two distinct lipid metabolism molecular subtypes in osteosarcoma patients, C1 and C2, with significantly different survival rates. C1 is characterized by increased cholesterol, fatty acid synthesis, and ketone metabolism. In contrast, C2 focuses on steroid hormone biosynthesis, arachidonic acid, and glycerolipid and linoleic acid metabolism. Feature selection in the TARGET-OS identified 12 lipid metabolism genes, leading to a model predicting osteosarcoma patient survival. The LMRS, based on the 12 identified genes, consistently accurately predicted prognosis across TARGET-OS, testing cohorts, and Meta-Cohort. Incorporating 12 published signatures, LMRS showed robust and significantly superior predictive capability. Our results offer a promising tool to enhance the clinical management of osteosarcoma, potentially leading to improved clinical outcomes.

## Introduction

Osteosarcoma (OS) is a type of bone cancer most commonly found in young people^1^. The incidence of osteosarcoma is estimated to be three cases per million of the population annually^2^. Chemotherapy and surgery are the primary treatment options for osteosarcoma patients^3^. However, many osteosarcoma individuals are either resistant to primary treatment or develop metastases, resulting in a 5-year survival rate of only 10–20%^4^. Currently, the most important biomarkers for clinical decision-making and risk classification are clinical features, such as metastasis. However, there was a significant difference in survival outcomes among patients with identical clinical features and therapeutic management. This study aims to identify the molecular subtypes of osteosarcoma, provide the appropriate treatment for each subtype, and improve the survival rates of these patients.

Lipids, as one of the most vital components of cells, are essential for signaling, preserving structural integrity, and storing energy^5^. The class encompasses a wide range of biomolecules, such as fatty acids (FAs), glycerides (neutral glycerides and phosphoglycerides), nonglyceride lipids (steroids and sphingolipids), and lipoproteins^6^. Lipid metabolism influences cancer cells through interactions with the tumor microenvironment and oncogenic signaling pathways. Crosstalk between lipid metabolism and the tumor microenvironment is essential in tumor immunogenicity by regulating the function of noncancer cells, especially immune-associated cells. For instance, lipid-laden dendritic cells were reported unable to present tumor-associated antigens^7^, and abnormal lipid accumulation suppressed dendritic cells' capacity to facilitate anti-tumor T cells^8^. Lipid metabolism could promote cancer stem cells by improving their ability to differentiate, migrate, and develop drug resistance in osteosarcoma^9^. Lipid metabolism was increased in malignant and metastatic osteosarcoma cells^10^. Besides, a previous study discovered that a critical class of lipids (diacylglycerols) is overexpressed in metastatic osteosarcoma cells compared to their nonmetastatic or nontumorigenic counterparts^11^. Inhibiting diacylglycerol synthesis could reduce the survivability and motility of OS cells.

High-throughput gene detection technologies have enabled the discovery of pivotal genetic features. Previous studies have tried to identify tumor subtypes by gene expression profiles^12^. Studies showed that even if tumor patients shared the same gender, age, and stage, they could still be classified into molecular subtypes with distinctive survival outcomes. Molecular subtype discovery could contribute to the development of personalized therapeutic strategies. Given the critical role of lipid metabolism in osteosarcoma development, there is an urgent need to investigate molecular subtypes of lipid metabolism in osteosarcoma patients.

This research focused on identifying osteosarcoma subtypes and forecasting patient outcomes by examining genes involved in lipid metabolism. Through feature selection, which involved identifying genes differentially expressed across subtypes, we could pinpoint essential genes for creating prognostic signatures. The expression profiles of these genes were then scrutinized within the TARGET-OS database to construct these prognostic signatures using gene expression data. The validity of these prognostic signatures was confirmed by testing them against independent datasets. These findings contribute significantly to improving risk stratification and enhancing the accuracy of predictions regarding patient prognosis in osteosarcoma cases.

## Materials and methods

### Data collection and processing

We obtained gene expression and survival profiles for osteosarcoma samples from the TARGET database by utilizing the TCGAbiolinks package, explicitly selecting the 'TARGET-OS' project^13^. Additionally, we identified three independent validation datasets for osteosarcoma: GSE21257^14^, GSE39058^15^, and GSE16091^16^, chosen for their availability of both gene expression and survival profiles. Following this preprocessing step, the resulting sample counts were 84 for TARGET-OS, 53 for GSE21257, 37 for GSE39058, and 34 for GSE16091. We opted for the median expression value when a gene had multiple values to ensure data consistency.

### Identification of molecular subtypes

#### Tumor lipid metabolism genes (TLMGs) from genecard

We conducted a search on the Genecard database (https://www.genecards.org) using the query term "[all] (tumor) AND [all] (lipid AND metabolism)" to identify genes related to lipid metabolism in the context of tumors^17^. We obtained a list of genes from the Genecard database, each associated with relevance score values. The relevance score is computed by considering the significance of various resources associating the gene with the disease. We set a threshold to focus our study on genes strongly connected to tumor lipid metabolism, opting only for genes with a relevance score exceeding 10. This filtering criterion led us to identify a subset of genes called Tumor Lipid Metabolism Genes (TLMGs). Our subsequent analysis was confined to TLMGs for which gene expression data were accessible in the TARGET-OS, GSE21257, GSE39058, and GSE16091 datasets. After this initial screening, we found 1,815 TLMGs had available gene expression values across these datasets.

#### Molecular subtype of osteosarcoma

To uncover distinct lipid metabolism patterns, we utilized unsupervised class discovery through the consensus clustering algorithm, facilitated by the "ConsensusClusterPlus" package^18^. This approach provides a solid basis for determining the optimal cluster number and composition, enhancing the precision of potential sample groupings. Notably, the sample sizes in the TARGET-OS, GSE21257, GSE39058, and GSE16091 datasets are relatively small, with the largest containing only 84 samples. To bolster the reliability of subtype identification, it was imperative to amalgamate samples from these four datasets into a single, unified dataset. However, directly merging these samples introduces the challenge of batch effects, which can skew the results. To mitigate this issue, we applied a binary transformation to the gene expression values within each cohort, categorizing them as 0 or 1 based on whether they were below or above the median expression level respectively. This step, known as binary transformation, has been proven to significantly minimize batch effects and is a well-adopted strategy in studies^19^. After this processing step, we aggregated the expression profiles from the TARGET-OS, GSE21257, GSE39058, and GSE16091 datasets into a unified Meta-Cohort. The clustering algorithm used the expression profiles of Tumor Lipid Metabolism Genes (TLMGs) within the Meta-Cohort, employing designated parameters: clusterAlg = "hc" for hierarchical clustering, and distance = "pearson" to measure correlation-based distances. To ensure the robustness of our classification, we executed the clustering process 500 times. This repetition aids in validating the stability and consistency of the identified clusters and determining the optimal number of molecular subtypes involved in thoroughly evaluating silhouette values, consensus matrices, delta area, and tracking plots.

#### The difference between molecular subtypes

To elucidate the survival variations among different molecular subtypes, we employed Kaplan–Meier survival curves, augmented with log-rank tests, to assess the survival discrepancies among these subtypes. Additionally, we utilized the Gene Set Variation Analysis (GSVA) package to compute enrichment scores about lipid metabolism pathways^20^. This method allowed for a detailed comparison of lipid metabolism scores across the molecular subtypes identified, thus providing insight into the unique biological functions and pathways defining each subtype. A prior study identified 114 metabolic pathways, of which 21 were dedicated to lipid metabolism, serving as a valuable resource for calculating the enrichment scores of lipid metabolism pathways^21^. Leveraging this information, we measured the activity levels of these 21 lipid metabolism pathways in two molecular subtype samples from our study. Such measurement facilitated the comparison of lipid metabolism between subtypes.

The pRRophetic package in R was employed to conduct an extensive drug sensitivity analysis, aimed at discerning differential sensitivities across two subtypes of osteosarcoma. This analysis facilitated the identification of drugs that exhibit variant responses among these subtypes, thereby assisting in selecting potential therapeutic agents tailored for specific osteosarcoma subtypes. The selection criteria were stringently set, focusing on drugs that showed a significant Benjamini & Hochberg adjusted *p*-value of < 0.05 and a notable group difference exceeding 0.2.

### Construction and validation of signature

#### Feature selection for signature

To address the issue of potential overfitting, we chose the TARGET-OS cohort from four cohorts to select the feature/gene. Firstly, we applied Fisher's exact test to analyze Tumor Lipid Metabolism Genes (TLMGs) between two subtypes, chosen for their suitability with small, binary datasets to identify genes with significant expression differences. Results were ranked by p-values and adjusted using the Benjamini–Hochberg method to reduce false positives, focusing on genes with adjusted p-values below 0.05 to denote differential expression. These genes, identified as differentially expressed genes (DEGs), were selected for further analysis. (2) We conducted univariate Cox proportional hazards analyses on the DEGs within the TARGET-OS cohort to evaluate their correlation with patient survival outcomes, identifying genes with notable prognostic significance. (3) We utilized the stepwise Akaike Information Criterion (stepAIC) approach to enhance our feature selection and mitigate overfitting risks. This method pinpointed the most prognostically informative genes incrementally, emphasizing model accuracy and simplicity. Through this process, we identified and selected pivotal genes for constructing our prognostic signature, prioritizing those offering the most substantial predictive value for survival in osteosarcoma patients.

#### Construction of lipid metabolism-related signature (LMRS)

To develop a lipid metabolism-related signature (LMRS) with enhanced accuracy and robustness, we leveraged the strengths of four distinct machine learning algorithms, creating ten different algorithmic configurations. This ensemble included four standalone algorithms—random survival forest (RSF), CoxBoost, generalized boosted regression models (GBM), and survival support vector machine (survival-SVM)—and six combined approaches integrating these techniques. This selection and combination of algorithms aimed to harness each method's unique predictive capabilities and strengths, thereby optimizing the predictive performance of the LMRS.

The development of the signature involved a comprehensive and sequential methodology. Initially, we applied ten distinct algorithm combinations to the selected prognostic genes from the step "2.3.1. Feature Selection for Signature ". This initial step was succeeded by a thorough assessment of each algorithm's performance across three distinct cohorts, specifically: (1) TARGET-OS, (2) a combined cohort of GSE21257, GSE39058, and GSE16091, and (3) the Meta-Cohort. This approach ensured an exhaustive evaluation across various cohorts. Due to the relatively limited sample sizes of GSE21257, GSE39058, and GSE16091, it was necessary to amalgamate these into a single cohort. This amalgamation was essential for accurately validating the Lipid Metabolism-Related Signature (LMRS) performance, as conducting fivefold cross-validation on each dataset separately would have resulted in errors from insufficient data. Notably, evaluating LMRS within the combined GSE cohort was critical because gene selection had been finalized in the TARGET-OS cohort, making GSE21257, GSE39058, and GSE16091 entirely independent datasets for testing LMRS. The conclusive phase of this process entailed the calculation of Harrell's Concordance Index (C-index) for each model across all validation datasets. The model demonstrating the highest C-index was subsequently identified as optimal.

#### Evaluation of the LMRS model

The optimal model, capable of producing a predictive probability, was utilized to define the LMRS score for each patient. Based on the median LMRS score, we computed these LMRS scores for patients across different cohorts and subsequently categorized them into two groups—high and low. This stratification validated the effectiveness of LMRS. To quantitatively evaluate the performance of the LMRS, we calculated the Area Under the Receiver Operating Characteristic Curve (AUC) values. These values measure the signature's ability to effectively discriminate between patients with different outcomes. Then, we conducted a log-rank test to rigorously assess the differences in overall survival between the high- and low- LMRS groups. This statistical test compares the survival distributions of two groups, allowing us to determine whether survival times are significantly different. The analysis was complemented by plotting survival curves, visually representing both groups' survival probability over time.

#### Comparison of LMRS with other signatures from publications

To benchmark the performance of the Lipid Metabolism-Related Signature (LMRS) performance against existing benchmarks, we conducted an exhaustive search for published signatures, culminating in the acquisition of 12 distinct signatures. These signatures were developed using a variety of algorithms, including stepwise Cox regression and Lasso regression, reflecting a broad spectrum of methodological approaches. Furthermore, these signatures encapsulate a wide range of biological processes, including but not limited to ferroptosis, stemness, fatty acid and lactate metabolism, aging, hexosamine biosynthesis, hypoxia, immune response, disulfidptosis, drug sensitivity, and oxidative stress. This diversity underscores the multifaceted nature of the biological mechanisms they represent. For each of these signatures, we meticulously calculated Harrell's concordance index (C-index) across all cohorts to gauge their predictive accuracy and effectiveness in prognostication. This comprehensive comparison highlights the relative performance of LMRS and showcases its potential advantages.

#### Relationship between LMRS scores and molecular subtypes

T-tests were used to compare the mean values of LMRS Scores between two molecular subtypes, identifying significant differences. Boxplots were then used to visually display the distribution of LMRS scores for each subtype. These plots show the median, variability, and any outliers in the LMRS scores, making it easier to see how the subtypes differ in terms of their LMRS distributions.

## Results

### Osteosarcoma molecular subtypes

**Figure 1:**
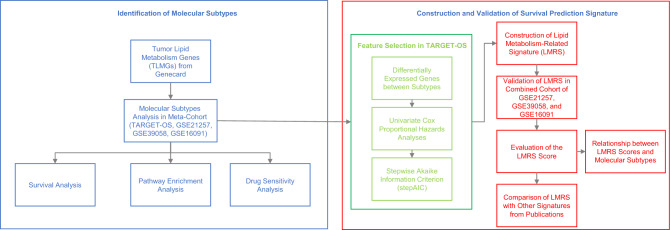
The workflow of this study.

The workflow of this study is shown in Fig. 1. For further analysis, we kept the samples with available expression data and survival information. Following this step, the resulting sample counts were 84 for TARGET-OS, 53 for GSE21257, 37 for GSE39058, and 34 for GSE16091. Through the search criterion "[all] (tumor) AND [all] (lipid AND metabolism)" in the GeneCards database, we got a list of genes, each assigned a relevance score. Selecting genes with an excellent relevance score of 10 yielded 2,759 genes associated with tumor lipid metabolism. Of these, 1,815 genes were represented with expression data across all datasets (TARGET-OS, GSE21257, GSE39058, and GSE16091). The expression data from these datasets were amalgamated into a Meta-Cohort, encompassing 208 osteosarcoma samples, with its clinical information detailed in Supplementary Table 1.

**Figure 2:**
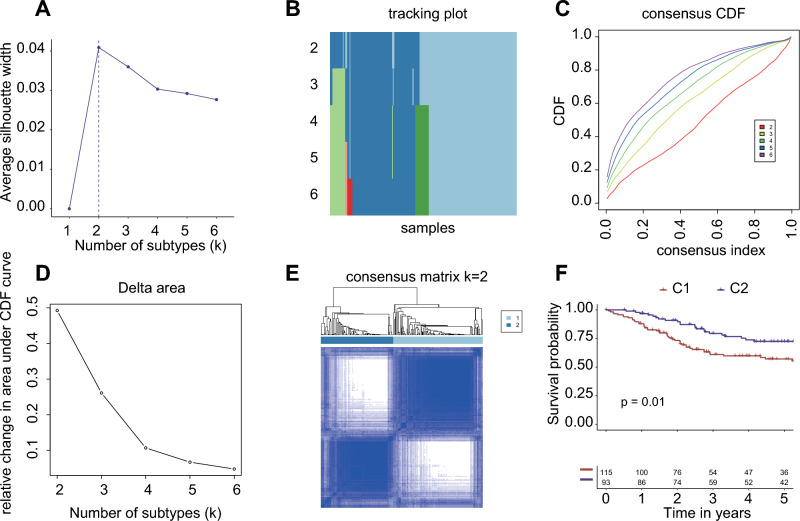
Meta-cohort osteosarcoma sample subtype consensus clustering. (**A**) The optimal number of subtypes is two, as indicated by the peak in silhouette width, which measures the similarity of samples within clusters. A higher silhouette width means more accurate clustering. (**B**) This plot depicts how samples are divided into subtypes, with the color intensity of each row showing the proportion of samples in each subtype. This plot helps track cluster changes over time, highlighting items frequently switching clusters as indicators of unstable cluster membership. (**C**) Demonstrates the cumulative distribution function (CDF) plot to identify the best number of subtypes by showing where the CDF curve peaks, indicating the highest clustering reliability. (**D**) Changes in the area under the CDF curve when comparing subtype counts (k) to k-1, showing the point of consensus maximization. This helps identify the optimal number of subtypes by finding where the increase in consensus becomes negligible, marking the most reliable clustering configuration. (**E**) The consensus matrices for two subtypes, with values ranging from 0 (no clustering) to 1 (perfect clustering), use color gradations to represent consensus levels. (**F**) Kaplan–Meier survival curves for the two molecular subtypes, offering insights into their prognostic significance.

Using expression data from 1815 genes, consensus clustering was conducted on the Meta-Cohort to identify molecular subtypes of osteosarcoma. The silhouette width plot (Fig. 2A) and tracking plot (Fig. 2B) confirmed 'two' as the optimal number of subtypes, despite the consensus cumulative distribution function (CDF) plot (Fig. 2C) and delta area plot (Fig. 2D) suggesting 'four' might be optimal. The decision to focus on two subtypes was based on two key considerations: (1) the tracking plot (Fig. 2B) indicated that most samples fell into two categories even when 'four' was considered optimal subtype number, and (2) a smaller number of subtypes is beneficial for developing machine learning prediction models, simplifying analysis and application. In the Meta-Cohort, there were 115 samples categorized under molecular subtype 1 (C1) and 93 samples under molecular subtype 2 (C2), as shown in Fig. 2E. The Kaplan–Meier survival analysis indicated that patients classified within C2 exhibited significantly longer survival time compared to those in C1, as depicted in Fig. 2F.

### The significant differences between molecular subtypes

We utilized the Gene Set Variation Analysis (GSVA) package to compute enrichment scores for 21 lipid metabolism pathways of samples in the Meta-Cohort. We plotted them using a heatmap (Supplementary Fig. 1). Among the 21 lipid metabolism pathways, 14 were found to be significantly different between the two subtypes by T-test. The C1 subtype is characterized by enhanced cholesterol, fatty acid biosynthesis and elongation, and ketone metabolism. In contrast, the C2 subtype is distinguished by its emphasis on steroid hormone (including aldosterone, cortisol, estradiol, and testosterone) biosynthesis, arachidonic acid metabolism, and glycerolipid and linoleic acid metabolism, indicating a more active role in lipid and hormone metabolism. Utilizing the pRRophetic package, we conducted a comprehensive analysis to quantify the differences in drug sensitivity between two distinct subtypes (Supplementary Table 2). This analysis uncovered that a total of 48 drugs exhibited heightened sensitivity towards the C1 subtype, characterized by lower IC50 values, indicating a more pronounced therapeutic response in this group. Conversely, a smaller subset of 5 drugs demonstrated greater sensitivity towards the C2 subtype, suggesting a tailored therapeutic potential for each subtype based on specific drug responses.

### Construction of a consensus signature

#### The feature selection process

Our research is dedicated to developing a dependable machine-learning model that avoids overfitting during its training phase. To achieve this, we limited the feature selection process to the TARGET-OS cohort, one among four cohorts studied. This selection involved a multifaceted pipeline that incorporated the analysis of differentially expressed genes (DEGs), univariate Cox analysis, and the stepwise Akaike Information Criterion (stepAIC) methodology, all aimed at identifying the most informative genes for constructing our model's signature. (1) Initially, we performed Fisher's exact test on all 1,815 Tumor Lipid Metabolism Genes (TLMGs) to distinguish between the two subtypes. By applying p-value adjustment, we identified 698 genes that showed significant differential expression in the TARGET-OS (adjusted *p*-value < 0.05). These DEGs were then subjected to further scrutiny. (2) Then, univariate Cox analysis of 698 DEGs enabled us to pinpoint 35 genes with prognostic value. This subset was further categorized based on the Cox model coefficient values, distinguishing 17 genes as protective (due to their negative coefficients) and 18 as risky (given their positive coefficients). (3) We employed the stepwise Akaike Information Criterion (stepAIC) approach to refine our gene selection. This iterative process, which evaluates the addition or removal of genes based on their effect on the Akaike Information Criterion, aims to strike an optimal balance between the model's complexity and explanatory power. Ultimately, this rigorous methodology led us to identify 12 genes as the most suitable for signature construction, marking a significant step forward in our model's development.

**Figure 3:**
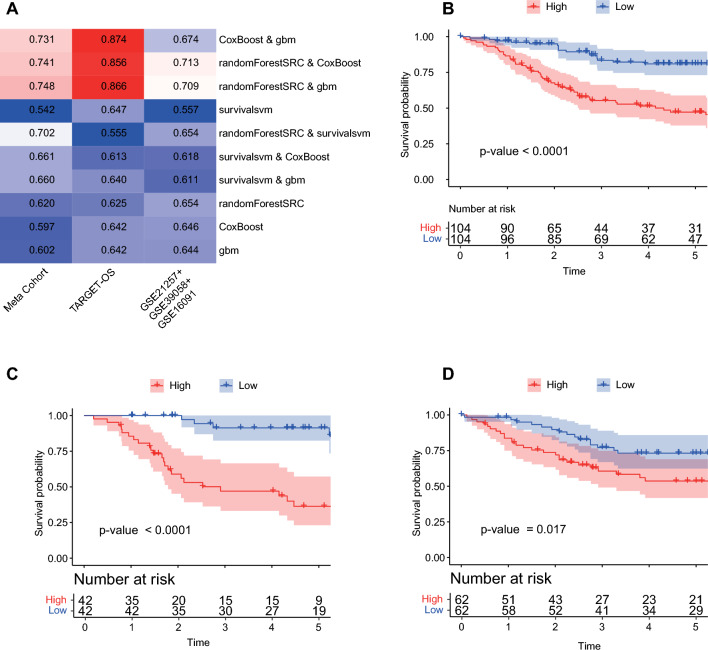
Analysis of Lipid Metabolism-Related Signature (LMRS) Performance Across Different Cohorts and Its Prognostic Value. (**A**) The efficacy of the Lipid Metabolism-Related Signature (LMRS) across various cohorts is quantified by the concordance index (C-index). Given the relatively small sample sizes of cohorts GSE21257, GSE39058, and GSE16091, these cohorts were amalgamated into a single cohort for validation. This approach was necessitated to ensure the robustness of LMRS validation, as executing fivefold cross-validation on each cohort failed from the limited data available. (**B**) Kaplan–Meier curves illustrating overall survival stratified by LMRS within the Meta-Cohort cohort. (**C**) Kaplan–Meier survival analysis of overall survival based on LMRS in TARGET-OS. (**D**) Kaplan–Meier curves for overall survival according to LMRS in the combined cohort comprising GSE21257, GSE39058, and GSE16091.

Following our analysis, 12 genes were further examined using a machine learning-based integrative approach, creating a Lipid Metabolism-Related Signature (LMRS). We implemented ten distinct prediction models within a fivefold cross-validation setup to evaluate our model's robustness and predictive performance. These models underwent rigorous testing across various cohorts to ensure a thorough evaluation. The cohorts examined included TARGET-OS (84 samples), a combination of three cohorts (GSE21257, GSE39058, GSE16091) totaling 124 samples, and a Meta-Cohort comprising 208 samples. The C-index was utilized to gauge the predictive accuracy of the ten models, with three machine-learning combinations displaying consistent precision across the datasets. Notably, the CoxBoost and GBM combination achieved a C-index of 0.874 within the TARGET-OS cohort, which is expected since the feature selection was conducted specifically within this cohort. Our signature also secured a C-index of 0.713 in the combined cohort of GSE21257, GSE39058, and GSE16091 through randomForestSRC & CoxBoost. The relatively small sample sizes of GSE21257, GSE39058, and GSE16091 necessitated their combination into a single cohort to validate the performance of LMRS, as performing fivefold cross-validation on each individually would lead to errors due to insufficient data. This combined cohort, independent of the feature selection process, served as an independent validation set for the machine learning models. The randomForestSRC & CoxBoost combination, yielding the highest average C-index of 0.713 (Fig. 3A), was identified as the most optimal model. This highlights the model's robustness and effectiveness in predicting outcomes across diverse datasets.

### Evaluation of the LMRS score

**Figure 4:**
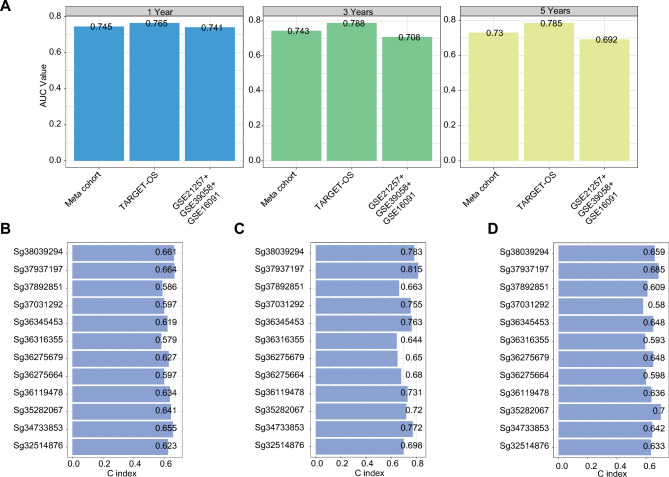
Robust performance of LMRS alongside comparisons of gene expression signatures. (**A**) Time-dependent ROC (Receiver Operating Characteristic) analysis depicts the efficacy of LMRS in predicting overall survival at 1, 3, and 5 years. (**B**) The C-index evaluation of 12 published signatures within the Meta-Cohort. (**C**) The C-index assessment of 12 published signatures within the TARGET-OS dataset. (**D**) The C-index analysis of the combined cohort of GSE21257, GSE39058, and GSE16091.

Next, an LMRS score value was calculated by the optimal model (randomForestSRC & CoxBoost) model, which demonstrated the highest C-index. All patients were assigned into high- and low-score groups according to the median value. As illustrated in Fig. 3B–D, patients in the high-score group had significantly dismal overall survival relative to the low-score group in the datasets of the Meta-Cohort (Fig. 3B), TARGET-OS cohort (Fig. 3C), and the combined cohort of GSE21257 + GSE39058 + GSE16091 (Fig. 3D; all p-value < 0.05). ROC analysis measured the discrimination of LMRS, with 1-, 3-, and 5-year AUCs of 0.745, 0.743, and 0.730 in Meta-Cohort, 0.765, 0.788, and 0.785 in TARGET-OS; 0.741, 0.708, and 0.692 in the combined cohort of GSE21257 + GSE39058 + GSE16091; respectively (Fig. 4A). All these indicators suggested that LMRS had stable and robust performance in multiple independent cohorts.

### Comparison of gene expression-based prognostic signatures

Recently, advancements in next-generation sequencing and big data technologies have led to the development of numerous prognostic and predictive gene expression signatures utilizing machine learning. To evaluate the performance of the Lipid Metabolism-Related Signature (LMRS) relative to other existing signatures, we methodically collected published signatures, including 12 distinct signatures (Table 1). These signatures are linked to various biological processes, including ferroptosis, cellular stemness, metabolism of fatty acids and lactate, aging, hexosamine biosynthesis, hypoxia, immune response, disulfidptosis, drug sensitivity, and oxidative stress. Following the same methodology described in section "Construction of lipid metabolism-related signature (LMRS)" for the construction of LMRS, we calculated the maximum Concordance Index (C-index) for each signature across all datasets using ten different machine learning models. LMRS achieved its highest C-index values of 0.748 in the Meta-Cohort, 0.874 in the TARGET-OS dataset, and 0.713 in a combined cohort (GSE21257, GSE39058, GSE16091). In comparison, the published signatures reached maximum C-index values of 0.664 in the Meta-Cohort (Fig. 4B), 0.815 in the TARGET-OS (Fig. 4C), and 0.700 in the combined cohort (GSE21257, GSE39058, GSE16091) (Fig. 4D). These findings underscore the superior performance and stability of LMRS across all evaluated datasets.

**Table 1:** The 12 signatures from published papers.

PMID	Genes	Citation
32,514,876	*KRT5, HIPK2, MAP3K5, CD5*	^22^
34,733,853	*DLL1, EOMES, ERCC2, FOLR1, MEF2C, PSMA5, PTN, SPI1*	^23^
35,282,067	*RP2, PHB, MYO6, MLH1, CSNK2B, RPL37A, CEBPA*	^24^
36,119,478	*SLC7A7, MYC, ACSS2*	^25^
36,275,664	*PRKACB, AIM1, EVI2B, TCEA3*	^26^
36,275,679	*GPI, PGM3, UAP1, OGT, MGEA5*	^27^
36,316,355	*SLC2A1, FBP1*	^28^
36,345,453	*IGF2, CTSO, NPC2, APBB1IP*	^29^
37,031,292	*WAS, CORT, WNT16, GLB1L2*	^30^
37,892,851	LRPPRC, MYH9	^31^
37,937,197	CPE, LASP1, PDK1, *LACTB*	^32^
38,039,294	ZYX, GJA5, GAL, GRAMD1B, CKMT2	^33^

### Relationship between LMRS scores and molecular subtypes

**Figure 5:**
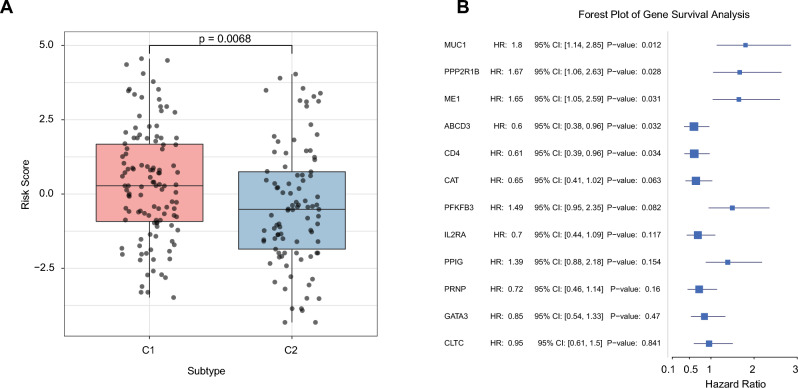
Analysis of LMRS Scores and Gene Survival Impact in Meta-Cohort. (**A**) Comparison of LMRS score distributions between samples C1 and C2 in the Meta-Cohort. (**B**) Univariate Cox regression analysis examining the association between gene expression levels and overall survival in the Meta-Cohort.

By T-test, we found that the C1 subtype has a significantly higher LMRS score than the C2 subtype (Fig. 5A). Univariate Cox analysis was done for each gene in the signature to determine the impact of the expression of 12 genes in LMRS. Patients with higher expression levels of *MUC1*, *PPP2R1B*, and *ME1* had a worse prognosis for survival than those with lower expression levels. Additionally, patients with higher expression levels of *ABCD3* and *CD4* were more likely to survive longer (Fig. 5B).

## Discussion

Lipid metabolism plays a critical role in diverse tumor properties, including tumorigenesis, invasion, and metastasis^34^. We applied consensus clustering algorithms to gene expression data focusing on lipid metabolism, successfully identifying two prognostic subtypes of osteosarcoma, labeled as C1 and C2. This distinction paves the way for more personalized approaches in osteosarcoma treatment. The C1 subtype is characterized by enhanced cholesterol and fatty acid biosynthesis, whereas the C2 subtype primarily involves steroid hormone biosynthesis. This highlights osteosarcoma's diverse and complex nature, offering new insights into potential therapeutic targets.

The incidence of osteosarcoma is estimated to be three cases per million of the population annually. This limits the number of available samples from being publicly available. For example, the datasets from TARGET-OS, GSE21257, GSE39058, and GSE16091 are characterized by their notably limited sample sizes, with the most extensive dataset comprising only 84 samples. This scarcity necessitated the consolidation of data from these sources into a comprehensive dataset to enhance the accuracy of subtype classification. However, such integration faces the obstacle of batch effects, potentially distorting the analysis. To address this, we introduced a binary transformation method for the gene expression data, assigning a value of 0 or 1 to indicate expression levels falling below or surpassing the median, respectively. This approach, known as binary transformation, effectively reduces batch-related discrepancies, representing a widely recognized method in research to ensure data consistency.

Our gene selection for cancer prognosis integrated a sophisticated approach that combines Fisher's exact test, univariate Cox proportional hazards analyses, and the stepAIC. This methodology is designed to identify genes with significant differential expression and predictive value and ensure the resulting prognostic model is both simple and accurate. By achieving a delicate balance between model complexity and predictive accuracy and carefully selecting a manageable set of clinically relevant genes, our approach transcends the capabilities of traditional algorithms like Random Forest, SVM, or LASSO. Our strategy emphasizes the importance of specificity in gene selection, the reduction of overfitting risks, and the enhancement of clinical applicability, offering a practical pathway for developing streamlined, cost-effective diagnostic tools in the context of cancer prognosis.

Computational methods have emerged as a potent tool for analyzing genomic data^35,36^. Our study incorporated four multicenter cohorts to elucidate the molecular distinctions across various prognostic subtypes and enhance their clinical relevance. We designated TARGET-OS as the primary discovery cohort for feature selection, while the remaining three served as independent validation cohorts. We identified the optimal Lipid Metabolism Related Signature (LMRS) by evaluating ten algorithm combinations to mitigate the biases inherent in algorithm selection. A significant challenge in biomedical model development with artificial intelligence and machine learning is overfitting, where models perform well on training data but falter on external validation sets. In our findings, a machine learning model combining CoxBoost and GBM demonstrated superior performance in the TARGET-OS discovery cohort, with a C-Index of 0.874, which, however, dropped to 0.674 in the validation cohorts. We prioritized the highest C-index from the independent testing cohorts to counteract overfitting as our selection criterion. By reducing redundant data through stepAIC, we successfully selected a 12-gene signature, LMRS, through meticulous feature selection. Kaplan–Meier analysis, ROC curves, and calibration plots all underscored the exceptional predictive accuracy of LMRS across the training, testing, and Meta-Cohort. Moreover, compared to 12 previously published signatures, LMRS consistently outperformed the others in nearly all cohorts, showcasing its robustness. This thorough evaluation confirms the strong prognostic potential of LMRS in various cohorts, setting it apart from existing models.

This study differed from previous studies in the following aspects. (1) We systematically collected four multicenter cohorts and selected the algorithm with the largest average C-index in the testing cohorts to construct our signature. (2) To prevent inappropriate modeling methods due to personal preference, we combined four recognized machine-learning algorithms into ten combinations and selected the best model based on their accuracy. While we have tried to be as rigorous and comprehensive as possible in our research, some limitations should be noted. This research has a few limitations that need to be addressed. Firstly, these 12 genes demonstrated their ability for osteosarcoma subtype identification and survival prediction; however, the study relied solely on genetic analysis, which is inadequate; experimental validation is required to support these findings and conclusions. Secondly, the osteosarcoma cohorts used in this study are from Norway and America. The geographic and racial variation among osteosarcoma patients may pose potential challenges in the application of our molecular subtype and survival model to Chinese patients. To address this problem, more osteosarcoma datasets from Chinese patients are required. In the future, we plan to validate the molecular subtype and survival model on Chinese patients through multicenter collaborations.

## Conclusion

The current study utilized consensus clustering to identify two distinct molecular subtypes of osteosarcoma based on tumor lipid metabolism genes. By a series of feature selection analyses, we have provided a signature that can accurately predict the survival outcome of osteosarcoma. Our research may provide novel insights into developing new medications targeting osteosarcoma, establish a theoretical basis for personalized treatment plans, and facilitate classifying individuals based on their molecular subtype.

### Supplementary Information

Supplementary Information 1.

Supplementary Information 2.

## Data Availability

The data used to support this study are downloaded from the publicly available databases (TRGET-OS and GEO), and their links were included within the article.
