# Supplementary material for: Machine learning survival prediction using tumor lipid metabolism genes for osteosarcoma
Source: Sci Rep. 2024 Jun 5;14:12934. doi: 10.1038/s41598-024-63736-y (PMC11153634; doi:10.1038/s41598-024-63736-y)
Supplement: Supplementary file 1 — Supplementary Information 1. [file 41598_2024_63736_MOESM1_ESM.pdf]

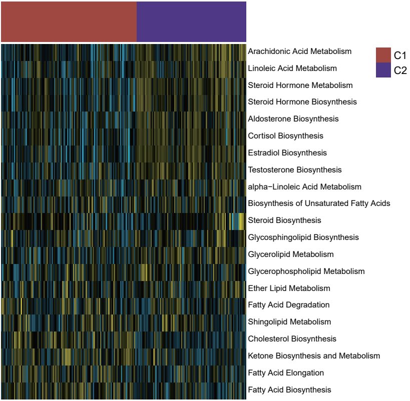

Supplementary Figure 1. The enrichment scores for 21 lipid metabolism pathways of samples in the Meta-Cohort.
