# Supplementary material for: Machine learning survival prediction using tumor lipid metabolism genes for osteosarcoma
Source: Sci Rep. 2024 Jun 5;14:12934. doi: 10.1038/s41598-024-63736-y (PMC11153634; doi:10.1038/s41598-024-63736-y)
Supplement: Supplementary file 2 — Supplementary Information 2. [file 41598_2024_63736_MOESM2_ESM.docx]

| sample | OS (0 for alive; 1 for dead) | OS Time (Year) |
| --- | --- | --- |
| TARGET_40_0A4HLD | 0 | 10.81 |
| TARGET_40_0A4HMC | 0 | 1.03 |
| TARGET_40_0A4HX8 | 0 | 4.79 |
| TARGET_40_0A4HXS | 0 | 8.08 |
| TARGET_40_0A4HY5 | 1 | 0.79 |
| TARGET_40_0A4I0Q | 1 | 1.42 |
| TARGET_40_0A4I0W | 0 | 1.87 |
| TARGET_40_0A4I3S | 0 | 5.05 |
| TARGET_40_0A4I42 | 1 | 1.7 |
| TARGET_40_0A4I48 | 0 | 4.43 |
| TARGET_40_0A4I4M | 0 | 1.94 |
| TARGET_40_0A4I4O | 1 | 1.72 |
| TARGET_40_0A4I5B | 0 | 1.7 |
| TARGET_40_0A4I65 | 0 | 16 |
| TARGET_40_0A4I6O | 1 | 5.22 |
| TARGET_40_0A4I8U | 0 | 2.69 |
| TARGET_40_0A4I9K | 0 | 6.18 |
| TARGET_40_PAKFVX | 0 | 2.53 |
| TARGET_40_PAKXLD | 0 | 6.75 |
| TARGET_40_PAKZZK | 1 | 4.15 |
| TARGET_40_PALECC | 1 | 2.07 |
| TARGET_40_PALFYN | 1 | 2.75 |
| TARGET_40_PALHRL | 0 | 11.98 |
| TARGET_40_PALKDP | 0 | 8.99 |
| TARGET_40_PALKGN | 0 | 5.81 |
| TARGET_40_PALWWX | 0 | 5.93 |
| TARGET_40_PALZGU | 1 | 7.9 |
| TARGET_40_PAMEKS | 1 | 2.35 |
| TARGET_40_PAMHLF | 0 | 5.24 |
| TARGET_40_PAMHYN | 0 | 10.68 |
| TARGET_40_PAMJXS | 1 | 10.58 |
| TARGET_40_PAMLKS | 1 | 0.49 |
| TARGET_40_PAMRHD | 1 | 0.2 |
| TARGET_40_PAMTCM | 0 | 8.22 |
| TARGET_40_PAMYYJ | 1 | 0.74 |
| TARGET_40_PANGPE | 0 | 9.25 |
| TARGET_40_PANGRW | 0 | 9.25 |
| TARGET_40_PANMIG | 1 | 2.13 |
| TARGET_40_PANPUM | 1 | 0.81 |
| TARGET_40_PANSEN | 0 | 6.18 |
| TARGET_40_PANVJJ | 0 | 8.48 |
| TARGET_40_PANXSC | 0 | 3.33 |
| TARGET_40_PANZHX | 0 | 3.62 |
| TARGET_40_PANZZJ | 0 | 4.21 |
| TARGET_40_PAPIJR | 0 | 4.71 |
| TARGET_40_PAPKWD | 1 | 1.16 |
| TARGET_40_PAPNVD | 1 | 2.53 |
| TARGET_40_PAPWWC | 0 | 6.9 |
| TARGET_40_PAPXGT | 0 | 6.92 |
| TARGET_40_PARBGW | 0 | 0.78 |
| TARGET_40_PARDAX | 1 | 1.86 |
| TARGET_40_PARFTG | 1 | 1.79 |
| TARGET_40_PARGTM | 0 | 7.15 |
| TARGET_40_PARJXU | 1 | 4.47 |
| TARGET_40_PARKAF | 1 | 2.91 |
| TARGET_40_PASEBY | 0 | 5.61 |
| TARGET_40_PASEFS | 0 | 5.79 |
| TARGET_40_PASFCV | 0 | 5.65 |
| TARGET_40_PASKZZ | 0 | 1.48 |
| TARGET_40_PASNZV | 0 | 4.79 |
| TARGET_40_PASRNE | 0 | 4.68 |
| TARGET_40_PASSLM | 0 | 5.11 |
| TARGET_40_PASUUH | 0 | 4.45 |
| TARGET_40_PASYUK | 0 | 5.12 |
| TARGET_40_PATAWV | 0 | 4.66 |
| TARGET_40_PATEEM | 0 | 3.98 |
| TARGET_40_PATJVI | 0 | 4.32 |
| TARGET_40_PATKSS | 1 | 2.08 |
| TARGET_40_PATMIF | 0 | 4.33 |
| TARGET_40_PATMPU | 1 | 4.33 |
| TARGET_40_PATMXR | 1 | 1.06 |
| TARGET_40_PATPBS | 0 | 4.02 |
| TARGET_40_PATUXZ | 1 | 1.4 |
| TARGET_40_PAUBIT | 0 | 2.82 |
| TARGET_40_PAUTWB | 0 | 1.88 |
| TARGET_40_PAUTYB | 1 | 1.47 |
| TARGET_40_PAUUML | 0 | 1.84 |
| TARGET_40_PAUVUL | 1 | 0.95 |
| TARGET_40_PAUXPZ | 1 | 1.66 |
| TARGET_40_PAUYTT | 0 | 1.59 |
| TARGET_40_PAVALD | 0 | 1.51 |
| TARGET_40_PAVCLP | 0 | 1.4 |
| TARGET_40_PAVDTY | 0 | 1.01 |
| TARGET_40_PAVECB | 0 | 1.3 |
| GSM530667 | 1 | 2.25 |
| GSM530899 | 1 | 1.75 |
| GSM531283 | 0 | 3.83 |
| GSM531284 | 0 | 2.33 |
| GSM531285 | 1 | 0.92 |
| GSM531286 | 0 | 3.08 |
| GSM531287 | 0 | 3.75 |
| GSM531288 | 1 | 1.08 |
| GSM531289 | 1 | 2.75 |
| GSM531290 | 0 | 2.08 |
| GSM531291 | 1 | 1.5 |
| GSM531292 | 1 | 2.5 |
| GSM531293 | 1 | 2.92 |
| GSM531294 | 1 | 0.33 |
| GSM531295 | 1 | 2.25 |
| GSM531296 | 0 | 2.17 |
| GSM531297 | 1 | 1.5 |
| GSM531298 | 1 | 15.75 |
| GSM531299 | 0 | 3 |
| GSM531300 | 0 | 10.25 |
| GSM531301 | 1 | 9.17 |
| GSM531302 | 0 | 5.25 |
| GSM531303 | 0 | 5 |
| GSM531304 | 0 | 5 |
| GSM531305 | 0 | 5 |
| GSM531306 | 1 | 0.83 |
| GSM531307 | 1 | 3.25 |
| GSM531308 | 0 | 7.92 |
| GSM531309 | 1 | 6.92 |
| GSM531310 | 0 | 20.5 |
| GSM531311 | 1 | 2.08 |
| GSM531312 | 1 | 3.33 |
| GSM531313 | 0 | 11.92 |
| GSM531314 | 1 | 0.92 |
| GSM531319 | 0 | 8.75 |
| GSM531320 | 0 | 6.5 |
| GSM531321 | 0 | 8.08 |
| GSM531322 | 1 | 2.75 |
| GSM531323 | 0 | 6.42 |
| GSM531324 | 1 | 3.92 |
| GSM531325 | 0 | 10 |
| GSM531326 | 0 | 7.58 |
| GSM531327 | 1 | 2.42 |
| GSM531328 | 0 | 2.67 |
| GSM531329 | 0 | 2.58 |
| GSM531330 | 1 | 2.08 |
| GSM531331 | 0 | 18.25 |
| GSM531332 | 0 | 16.08 |
| GSM531333 | 0 | 15.33 |
| GSM531334 | 0 | 16.17 |
| GSM531335 | 0 | 7.83 |
| GSM531351 | 0 | 7.25 |
| GSM531352 | 0 | 5 |
| GSM954790 | 1 | 10.53 |
| GSM954791 | 0 | 1.62 |
| GSM954792 | 0 | 6.88 |
| GSM954793 | 0 | 5.09 |
| GSM954794 | 0 | 2.53 |
| GSM954795 | 0 | 16.74 |
| GSM954796 | 0 | 2.35 |
| GSM954797 | 1 | 12.58 |
| GSM954798 | 0 | 2.87 |
| GSM954799 | 0 | 0.78 |
| GSM954800 | 0 | 4.17 |
| GSM954801 | 1 | 1.07 |
| GSM954802 | 0 | 16.34 |
| GSM954803 | 0 | 2.79 |
| GSM954804 | 0 | 10.36 |
| GSM954805 | 0 | 5.47 |
| GSM954806 | 0 | 4.93 |
| GSM954807 | 0 | 4.2 |
| GSM954808 | 0 | 6.22 |
| GSM954809 | 0 | 5.38 |
| GSM954810 | 0 | 5.91 |
| GSM954811 | 1 | 2.49 |
| GSM954812 | 0 | 4.63 |
| GSM954813 | 0 | 6.01 |
| GSM954814 | 0 | 1.18 |
| GSM954815 | 0 | 5.99 |
| GSM954816 | 1 | 2.01 |
| GSM954817 | 1 | 0.74 |
| GSM954818 | 1 | 1.18 |
| GSM954819 | 1 | 1.17 |
| GSM954820 | 0 | 2.33 |
| GSM954821 | 0 | 0 |
| GSM954822 | 0 | 1.12 |
| GSM954823 | 0 | 0.51 |
| GSM954824 | 1 | 2.08 |
| GSM954825 | 1 | 0.22 |
| GSM954826 | 0 | 2.67 |
| GSM402718 | 1 | 5.66 |
| GSM402719 | 0 | 7.95 |
| GSM402720 | 1 | 1.71 |
| GSM402721 | 0 | 9.05 |
| GSM402722 | 1 | 0.48 |
| GSM402723 | 1 | 1.32 |
| GSM402724 | 0 | 4.82 |
| GSM402725 | 1 | 3.8 |
| GSM402726 | 0 | 4.62 |
| GSM402727 | 1 | 2.9 |
| GSM402728 | 0 | 2.81 |
| GSM402729 | 0 | 3.35 |
| GSM402730 | 0 | 0.96 |
| GSM402731 | 1 | 1.06 |
| GSM402732 | 0 | 4.57 |
| GSM402733 | 0 | 2.54 |
| GSM402734 | 0 | 5.12 |
| GSM402735 | 0 | 3.93 |
| GSM402736 | 0 | 8.77 |
| GSM402737 | 1 | 0.12 |
| GSM402738 | 0 | 11.39 |
| GSM402739 | 0 | 9.79 |
| GSM402740 | 1 | 0.56 |
| GSM402741 | 0 | 7.77 |
| GSM402742 | 1 | 0.07 |
| GSM402743 | 1 | 0.62 |
| GSM402744 | 1 | 2.76 |
| GSM402745 | 0 | 13.01 |
| GSM402746 | 0 | 12.54 |
| GSM402747 | 1 | 10.28 |
| GSM402748 | 1 | 1.93 |
| GSM402749 | 0 | 13.25 |
| GSM402750 | 1 | 3.3 |
| GSM402751 | 0 | 12.43 |

**Supplementary Table 1.** The clinical information of samples in Meta-Cohort.

| drug | Mean value in C1 | Mean value in C2 | Adjusted p-value |
| --- | --- | --- | --- |
| piperlongumine | 1.438502 | 1.639552 | <0.01 |
| Dasatinib | 0.437595 | 0.641918 | <0.01 |
| QL.X.138 | 1.399108 | 1.603498 | <0.01 |
| CX.5461 | 3.05695 | 3.261723 | <0.01 |
| AUY922 | -3.30183 | -3.09574 | <0.01 |
| rTRAIL | -0.65609 | -0.44883 | <0.01 |
| PF.562271 | 1.769607 | 1.977249 | <0.01 |
| GSK.650394 | 3.220619 | 3.431385 | <0.01 |
| GSK429286A | 4.811641 | 5.02346 | <0.01 |
| Midostaurin | -0.16164 | 0.050482 | <0.01 |
| AG.014699 | 3.389034 | 3.602291 | <0.01 |
| TAE684 | 0.751947 | 0.967942 | <0.01 |
| ZG.10 | 1.24797 | 1.464205 | <0.01 |
| CI.1040 | 2.444929 | 2.227274 | <0.01 |
| Cisplatin | 2.771601 | 2.994547 | <0.01 |
| Dabrafenib | 3.68913 | 3.461357 | <0.01 |
| BMS.509744 | 2.77597 | 3.008093 | <0.01 |
| QL.VIII.58 | -1.38299 | -1.14804 | <0.01 |
| A.770041 | 2.133706 | 2.378472 | <0.01 |
| Genentech.Cpd.10 | 2.399032 | 2.647716 | <0.01 |
| Nutlin.3a.... | 3.763052 | 3.511748 | <0.01 |
| VX.680 | 1.003036 | 1.255751 | <0.01 |
| WZ3105 | -0.06703 | 0.191605 | <0.01 |
| AT.7519 | 0.951724 | 1.210823 | <0.01 |
| YK.4.279 | 1.236843 | 1.496017 | <0.01 |
| TW.37 | -0.72249 | -0.44636 | <0.01 |
| NPK76.II.72.1 | 2.811291 | 3.091726 | <0.01 |
| Vinblastine | -4.31786 | -4.03568 | <0.01 |
| Obatoclax.Mesylate | -1.17647 | -0.88441 | <0.01 |
| BX.912 | 2.796967 | 3.094497 | <0.01 |
| Navitoclax | 1.62685 | 1.328821 | <0.01 |
| JW.7.52.1 | -2.04054 | -1.7388 | <0.01 |
| BX.795 | 1.723738 | 2.030028 | <0.01 |
| Ispinesib.Mesylate | -1.97045 | -1.64578 | <0.01 |
| Paclitaxel | -3.53648 | -3.20904 | <0.01 |
| Trametinib | -0.74192 | -1.07328 | <0.01 |
| FK866 | -3.12922 | -2.78236 | <0.01 |
| Pyrimethamine | 3.527648 | 3.88817 | <0.01 |
| PHA.793887 | 2.335702 | 2.702988 | <0.01 |
| Vinorelbine | -4.20383 | -3.83035 | <0.01 |
| Cytarabine | 0.033277 | 0.408334 | <0.01 |
| Epothilone.B | -5.24249 | -4.86497 | <0.01 |
| Elesclomol | -3.33413 | -2.94846 | <0.01 |
| Bleomycin..50.uM. | 2.171003 | 2.557788 | <0.01 |
| X17.AAG | -0.74866 | -0.33508 | <0.01 |
| Talazoparib | 2.060321 | 2.506698 | <0.01 |
| BI.2536 | -2.38316 | -1.93615 | <0.01 |
| GW843682X | -3.01663 | -2.56896 | <0.01 |
| Etoposide | 1.237811 | 1.691939 | <0.01 |
| Gemcitabine | -2.87004 | -2.39843 | <0.01 |
| MLN4924 | -0.06243 | 0.423893 | <0.01 |
| Docetaxel | -5.21155 | -4.71304 | <0.01 |
| Thapsigargin | -4.38947 | -3.88326 | <0.01 |

**Supplementary Table 2.** The differences in drug sensitivity values between two distinct subtypes. A lower value indicates a more pronounced therapeutic response in this subtype.
